# Return of the moth: rethinking the effect of climate on insect outbreaks

**DOI:** 10.1007/s00442-019-04585-9

**Published:** 2020-01-09

**Authors:** Ulf Büntgen, Andrew Liebhold, Daniel Nievergelt, Beat Wermelinger, Alain Roques, Frederick Reinig, Paul J. Krusic, Alma Piermattei, Simon Egli, Paolo Cherubini, Jan Esper

**Affiliations:** 1 Department of Geography, University of Cambridge, Cambridge, CB2 3EN UK; 2 Swiss Federal Research Institute WSL, 8903 Birmensdorf, Switzerland; 3 Global Change Research Institute of the Czech Academy of Sciences (CzechGlobe), Department of Geography, Faculty of Science, Masaryk University, 613 00 Brno, Czech Republic; 4 USDA Forest Service Northern Research Station, Morgantown, WV 26505 USA; 5 Czech University of Life Sciences Prague, Forestry and Wood Sciences, 165 21 Prague, Czech Republic; 6 INRA, UR633 Unité de Recherche de Zoologie Forestière, Orléans, 45075 France; 7 Department of Geography, Johannes Gutenberg University, 55099 Mainz, Germany

**Keywords:** European Alps, Dendroecology, Insect outbreaks, North Atlantic Oscillation, Population cycles, *Zeiraphera diniana* or *griseana*

## Abstract

The sudden interruption of recurring larch budmoth (LBM; *Zeiraphera diniana* or *griseana* Gn.) outbreaks across the European Alps after 1982 was surprising, because populations had regularly oscillated every 8–9 years for the past 1200 years or more. Although ecophysiological evidence was limited and underlying processes remained uncertain, climate change has been indicated as a possible driver of this disruption. An unexpected, recent return of LBM population peaks in 2017 and 2018 provides insight into this insect’s climate sensitivity. Here, we combine meteorological and dendrochronological data to explore the influence of temperature variation and atmospheric circulation on cyclic LBM outbreaks since the early 1950s. Anomalous cold European winters, associated with a persistent negative phase of the North Atlantic Oscillation, coincide with four consecutive epidemics between 1953 and 1982, and any of three warming-induced mechanisms could explain the system’s failure thereafter: (1) high egg mortality, (2) asynchrony between egg hatch and foliage growth, and (3) upward shifts of outbreak epicentres. In demonstrating that LBM populations continued to oscillate every 8–9 years at sub-outbreak levels, this study emphasizes the relevance of winter temperatures on trophic interactions between insects and their host trees, as well as the importance of separating natural from anthropogenic climate forcing on population behaviour.

**Electronic supplementary material:**

The online version of this article (10.1007/s00442-019-04585-9) contains supplementary material, which is available to authorized users.

## Introduction

Periodic larch budmoth (LBM; *Zeiraphera diniana* or *griseana* Gn.) outbreaks are a classic example of population cycles (Baltensweiler and Rubli 1999), with densities regularly oscillating every 8–9 years, ranging from ~ 1 to 20,000 larvae per host tree (*Larix decidua* Mill.). Recurring LBM epidemics, within an altitudinal range of ~ 1700 to 2000 m asl (Johnson et al. 2010), have been reconstructed without clear interruption over several centuries across the Alpine arc (Büntgen et al. 2009; Hartl-Meier et al. 2017; Kress et al. 2009; Nola et al. 2006; Rolland et al. 2001; Saulnier et al. 2017), and back to mediaeval times in the Swiss Lötschental (Esper et al. 2007). Periodic outbreaks of this foliage-feeding Lepidoptera species can affect the functioning and productivity of larch forest ecosystems (Berryman 2002), with an estimated reduction in aboveground biomass of > 1100 kg ha^−1^ in the first 3–4 years after forest defoliation (Peters et al. 2017). Geographical patterns of twentieth century LBM outbreaks across the Alpine arc indicate recurring outbreaks likely appear as eastward travelling waves from western epicentres in the southern French Alps to subalpine regions in central and eastern Austria (Bjørnstad et al. 2002; Johnson et al. 2004). Despite extensive demographic studies of LBM oscillations since the mid-twentieth century (Baltensweiler et al. 1977), and comprehensive tree ring-based evidence of historical population peaks during the past three centuries (Büntgen et al. 2009), the relationship between biotic and abiotic factors responsible for the system’s stable periodicity is not fully understood (Baltensweiler and Rubli 1999; Turchin et al. 2003).

The unprecedented disappearance of widespread LBM forest defoliation since the late-1980s was unexpected (Wermelinger et al. 2018). While the hiatus of three Alpine-wide outbreaks from circa 1990 to 2010 may have afforded aesthetic benefits to residents and possibly even benefitted tourism (Wermelinger et al. 2018), the system’s failure has been interpreted as an example of an extraordinary breakdown of ecological behaviour in response to global warming (Esper et al. 2007; Ims et al. 2008). However, it remains unclear how, if at all, climate affects the intensity, frequency, and persistence of cyclic LBM population outbreaks. Though still debatable, previous work suggests increased winter temperatures lead to decreased egg survival during diapause (Baltensweiler 1993), and the temporal offset between larval and foliar development may dampen LBM population growth (Benz 1974; Wermelinger et al. 2018). Moreover, it has been argued that rising temperatures can shift the insect’s outbreak epicentres to higher elevations (Johnson et al. 2010). As climates warm, conditions for optimal LBM growth may move upwards, above the current distribution of dense and widespread subalpine larch forests, where sufficient foliage resources are lacking (Hartl-Meier et al. 2017). Despite numerous studies on a variety of biotic and abiotic factors that potentially influence the LBM system (Büntgen et al. 2009; Hartl-Meier et al. 2017; Johnson et al. 2010; Kress et al. 2009; Nola et al. 2006; Rolland et al. 2001; Saulnier et al. 2017), the identity of climatic controls on the sudden ‘collapse’ of widespread LBM outbreaks after 1982 remains a mystery.

Here, we combine high-resolution meteorological and dendrochronological measurements to report on the unexpected recent return of LBM outbreaks in 2017 and 2018 at different sites of the Alpine arc. We argue that even though widespread insect defoliation of subalpine larch trees across the European Alps has been absent for nearly 4 decades, LBM populations in this region most likely continued to oscillate every 8–9 years at sub-outbreak levels. Moreover, we suggest the current LBM outbreak conceivably occurred at higher elevations, and that the period, during which previous LBM outbreaks were lacking since 1982, was characterised by a climatic regime shift over the North Atlantic/European sector that resulted in particularly warm winters. Our study not only tests and supports the three interrelated theories of warming-induced elevational range shifts, phenological mismatch and trophic disruption, but also implicates the ecophysiological importance of winter temperatures for forest insect population outbreaks.

## Materials and methods

### Insect outbreak data

**Fig. 1:**
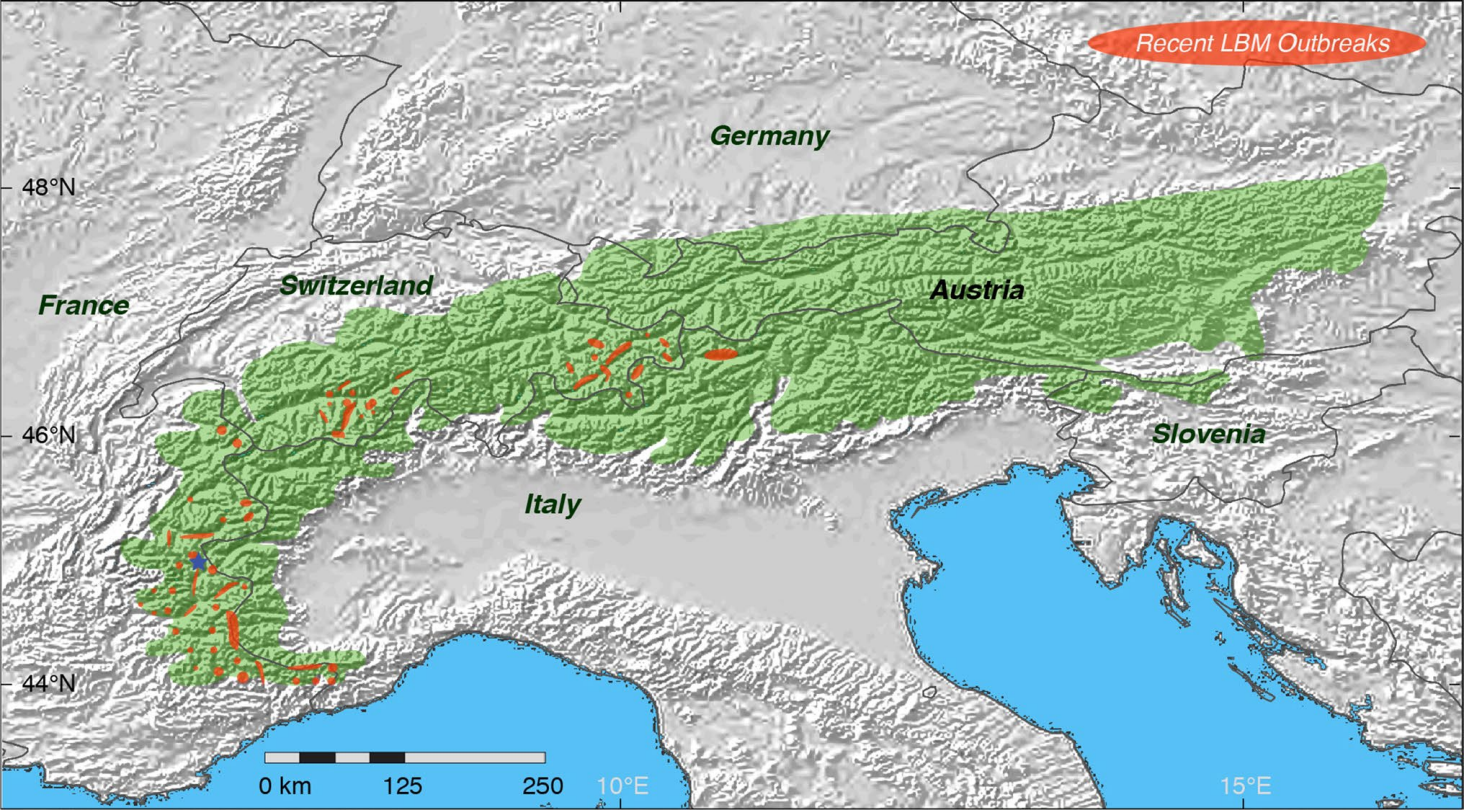
Spatial distribution of recent LBM outbreaks. Green shading depicts the potential (not actual) range of larch (*Larix decidua* Mill.) along the Alpine arc (Bjørnstad et al. 2002; Johnson et al. 2004), including subalpine forests in France, Italy, Switzerland, Germany, Austria and Slovenia. Orange areas approximate the location of defoliated subalpine larch stands in 2016–18, and the blue star refers to the permanent LBM plot at ‘Les Combes’ in the southern French Alps

Information on historical LBM outbreaks between 1950 and 1992 was obtained from Alpine-wide field observations of defoliation and a network of field locations where larval counts were made annually (Baltensweiler and Rubli 1999). Additional observations of defoliation since 2015 allowed mapping the most recent LBM outbreaks in Switzerland, France and Italy. Moreover, LBM larval density was estimated yearly since 1960 via branch sampling in a permanent plot at ‘Les Combes’, which is located at 1800 m asl in the southern French Alps (Fig. 1). Data at this site were collected using the prognosis method defined by Roques and Goussard (1982). From early to mid-June, once the first larval sheaths typical of 4th-instar LBM larvae were observed, five 40 cm-long branches were randomly cut per tree at mid-crown on ten trees selected according to a zig–zag path covering the entire plot. All larval sheaths were dissected, larvae were identified to species, and the LBM mean density per meter of branch was calculated. The ‘Les Combes’ plot is located on a sunny, south facing slope in the Briançon. The local LBM population cycle is, therefore, expected to be advanced by 1 year compared to other populations in the surrounding valleys in the southern French Alps. Thus, information from this plot is used in an extrapolative way to predict the development of LBM populations the following year(s) in the wider region. It should be noted in this regard, that the French study site ‘Les Combes’ is unique in its continuity and level of observational details. While first signs of defoliation and discoloration were reported in France since 2015 and in Switzerland since 2017, the appearance of symptoms in more eastern Alpine countries such as Austria and Slovenia would be expected to be delayed by 1–2 years (Bjørnstad et al. 2002; Johnson et al. 2004; Wermelinger et al. 2018). Locations of recent observations of new LBM defoliation were overlaid with GIS data describing the potential, natural distribution of larch (Caudullo et al. 2017; EUFORGEN 2018).

To investigate the timing and magnitude of past LBM defoliation impacts on larch growth, incremental measurements of wood production were obtained using a combination of dendrochronological and wood anatomical methods (Büntgen 2019). After the 2017 growing season (20-Sep-2017), 23 individual larch trees from around 1900 m asl, on the north-exposed slope of the Swiss inner-alpine Lötschental were sampled (Esper et al. 2007; Peters et al. 2017). From one core (tree 04b), a continuous thin section of 10–25 µm was cut with a sliding microtome (Gärtner and Nievergelt 2010). A combination of water and pure alcohol was used to moisten the cross-sectional surface, and a drop of glycerol was applied to prevent drying. The sample was bleached for 5 min with Eau de Javel (sodium hypochlorite and potassium hypochlorite), then double stained with a 1:1 Safranin and Astra-Blue solution that colours lignified structures red and non-lignified material blue (Gärtner and Schweingruber 2013). Surplus stain was removed with ethanol to further dehydrate the thin sections, which we finally embedded in Canada balsam under a permanent cover glass and dried for 12–24 h at 60 °C. All remaining larch samples were mounted and polished with sand paper of progressively finer grain size up to 800 grit. Ring width of these samples were measured on a Velmex Tree Ring Measuring System with a resolution of 0.001 mm (Velmex Inc., Bloomfield, NY, USA).

### Climate response analysis

Monthly mean temperatures (as well as the corresponding maximum and minimum values) and monthly precipitation totals were compiled from a high-resolution, gridded (0.25° × 0.25°) dataset of surface measurements (E-OBS v17.0) (Haylock et al. 2008), which were averaged over the greater study area (6–12° East and 46–47° North). Measurements from local meteorological stations in the eastern Swiss Alps were used for validation of the gridded product (Büntgen et al. 2014). Monthly mean temperatures, recorded at high-elevation stations in Grisons, were strongly correlated with the temperature indices from the nearest E-OBS grid cell (*r* > 0.9). A tipping- or breakpoint timeseries analysis that identifies periods at which statistical properties and patterns of a timeseries change (Rodionov 2004), was applied on the individual meteorological data for detecting possible regime shifts. The breakpoint test is a sequential version of the cumulative deviations of the means test (Rebstock 2002), combined with the *t* test (Rodionov 2004). One can think of it more simply as a sliding *t* test able to detect multiple inflection points in a timeseries. The gridded temperature datasets, as well as the monthly resolved indices of the North Atlantic Oscillation (NAO; Hurrell 1995) were used for comparison with LBM population dynamics since 1950. The NAO is a major source of seasonal to interdecadal variability in the Earth’s climate system (Hurrell 1995), and its climate effect is most pronounced in winter. A positive winter index (NAO +) produces increased westerlies and, consequently, mild and wet November–February conditions in central Europe, including the Alpine arc. In contrast, when the index is low (NAO−), westerlies are suppressed, and much of central Europe is dominated by cold and dry winters. However, a strong high-pressure influence from the east can result in a persistent blocking of westerly zonal flow. The NAO indices were extracted from NOAA’s Climate Prediction Centre (CPC; Chen and van den Dool 2003). Phenological observations of larch foliage expansion were extracted from four high-elevation sites in the Swiss Alps between 1500 and 1800 m asl (Davos, Lenzerheide, Pontresina and St. Moritz). It should be noted that historical long-term observations of larch and LBM phenology originated from the host dominated subalpine forest belt in the eastern Swiss Alps, which has been identified previously as a key biogeographic hotspot for cyclic LBM outbreaks (Baltensweiler and Rubli 1999). This region has further been the geographical focus of considerable LBM population research in the twentieth century (see Baltensweiler et al. 2008 and references therein). All continuous timeseries were expressed as day of the year (DOY). Though encompassing periods both with and without LBM outbreaks, the temporal availability of precise LBM and meteorological data restricts our analyses to post-1950.

## Results

**Fig. 2:**
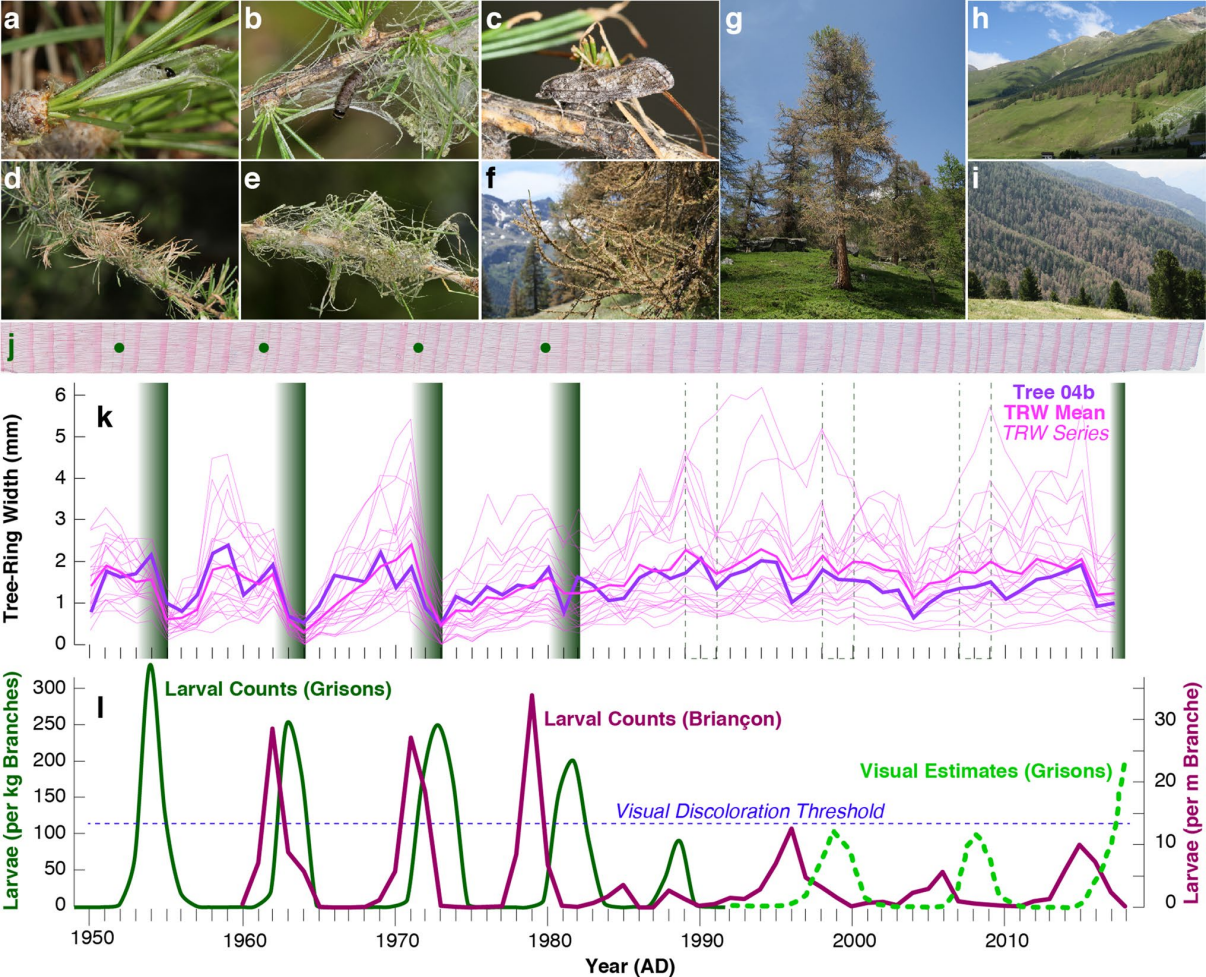
Ecophysiological and wood anatomical characteristics of LBM outbreaks. **a**–**c** Photos of different insect stages, including early and late instar larvae as well as an adult moth. **d**–**f** Sequence of infested larch needles, shoots and branches with silk webs. **g**–**i** Various defoliation levels ranging from single larch trees to entire mountainsides. All photos were taken from 19-Jun-2018 to 12-Jul-2018 at different sites in the eastern and western Swiss Alps in Grisons and Valais, respectively (photos BW). **j** High-resolution, thin section image of a larch tree-ring core sample (1950–2017) from 1900 m asl in the Lötschental, Valais (tree04b), which was extracted 20-Sep-2017. **k** Tree-ring width variations of 23 larch samples from 1900 m asl in the Lötschental, Valais. The samples were extracted on 20-Sep-2017 and cover the period 1950–2017. The shaded green vertical bars refer to the four Alpine-wide, high-peak LBM outbreaks during 1953–55, 62–64, 71–73 and 80–82, whereas the dashed frames indicate three LBM cycles without substantial defoliation (1989–91, 1998–00 and 2007–09). l, Cyclic, 8.5-year LBM population dynamics as measured and estimated since 1950 in the eastern Swiss Alps (Grisons) (Baltensweiler and Rubli 1999; Wermelinger et al. 2018), superimposed on yearly counts of LBM larval density since 1960 in a single permanent plot at 1800 m asl in the southern French Alps (Les Combes, Briançon) (Roques and Goussard 1982)

Since 2016, there have been several local- to regional-scale observations of LBM outbreak-induced discoloration made by forest agencies and by the authors of this study (Fig. 1). Together with in situ larval counts in France, the most recent LBM epidemic in Switzerland and parts of the Italian Alps provides new insight into the ecological boundary conditions of high LBM population growth. The geographical extent of defoliation during the recent LBM outbreak is documented in the eastern (Grisons) and western (Valais) Swiss Alps (Figs. 1, 2a–i), though not yet reconstructed from tree-ring width chronologies (Fig. 2j–l). Interestingly, the timing of the ongoing LBM epidemic matches with what would be anticipated based upon the system’s exceptional long-term regularity (Fig. 2j–l).

**Fig. 3:**
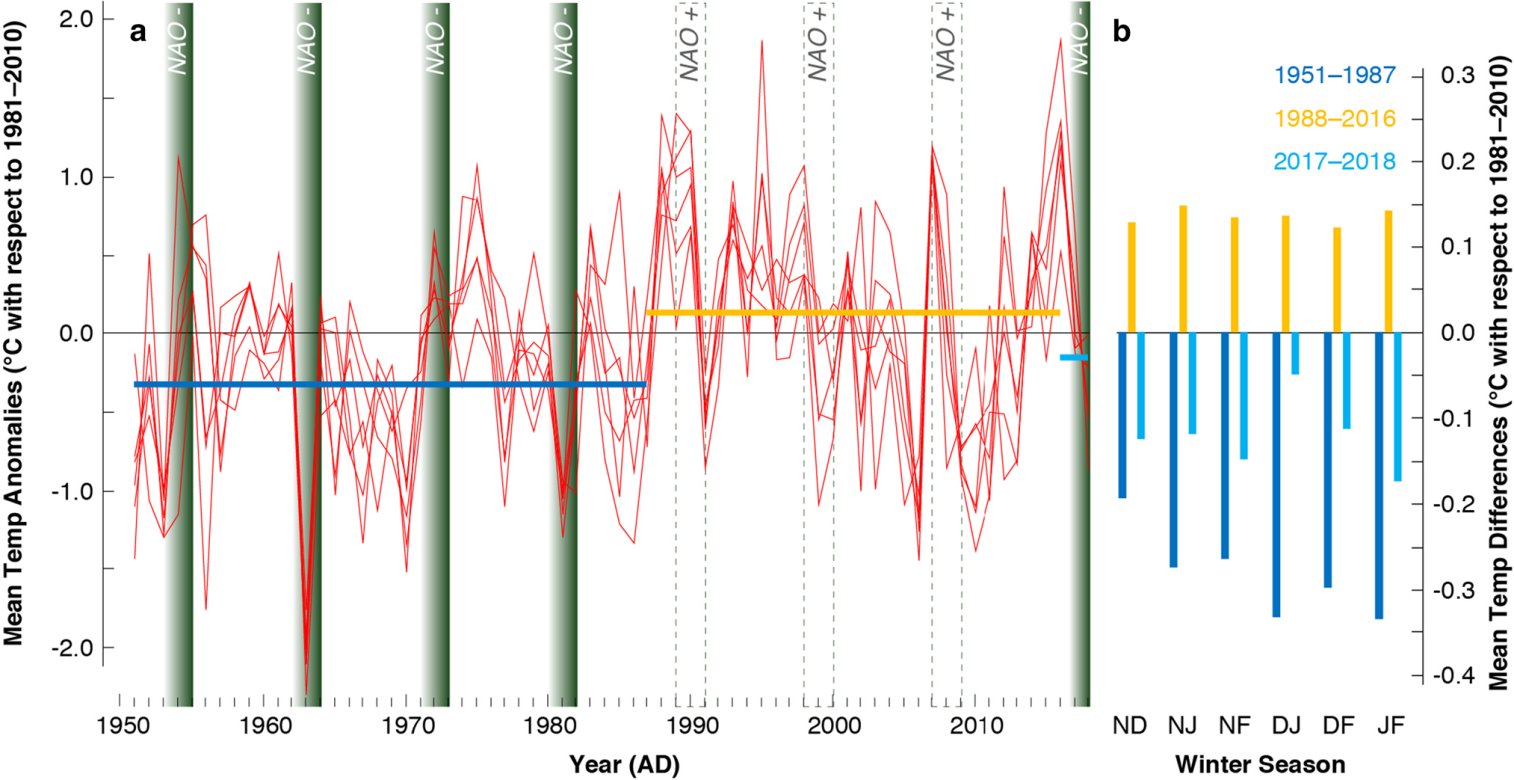
Temperature variability and LBM activity. **a** A total of six different combinations of monthly mean winter temperatures between November and February (Nov–Dec, Nov–Jan, Nov–Feb, Dec–Jan, Dec–Feb, Jan–Feb), expressed as anomalies from the 1981–2010 reference period (see Fig. S1 for a comparison with minimum and maximum temperatures). Superimposed on the vertical LBM cycles are the corresponding NAO phases. **b** Differences in mean winter temperatures between three periods of intense (1951–1987), absent (1988–2016), and returning (2017–2018) LBM outbreaks

Comparison of the four most severe and best documented Alpine-wide outbreaks between 1953 and 1982 against monthly resolved and spatially detailed temperature indices offers a better understanding of the climate sensitivity of the LBM system. Data on historical LBM population dynamics come from intensive sampling of larval counts and mapped forest discoloration measurements (Baltensweiler and Rubli 1999), as well as from synchronised negative anomalies in the ring width and latewood density of thousands of subalpine larch trees (Büntgen et al. 2009), and this study (Fig. 2k). Six different monthly combinations of mean winter temperatures between November and February produce strong negative anomalies, relative to the 1981–2010 reference period, during the four LBM outbreaks in 1953–1955, 1962–1964, 1971–1973 and 1980–1982 (Fig. 3a). Regardless of the window length, from 8 to 22 years, a statistically significant difference in the average temperatures was detected between 1984 and 1985 (*p* < 0.05). This observation is further corroborated when testing the seasonal means pre-1985, against those post-1985. Comparing the pre- and post-1985 averages with a one-tail *t* test [Ho: $\bar{\varvec{x}}$ (1985–2016) > $\bar{\varvec{x}}$ (1951–1984)], we find for all but one season (Jan–Feb) the post-1984 means are greater than the pre-1985 means (*p* < 0.05) (Table 1).

**Table 1:** LBM outbreaks during colder winter climates

	Nov–Dec	Nov–Jan	Nov–Feb	Dec–Jan	Dec–Feb	Jan–Feb	Mean Fig. 3
1951–1984	− 0.2298	− 0.2677	− 0.2507	− 0.3189	− 0.2792	− 0.2716	− 0.2696
1985–2016	0.1386	0.1014	0.0848	0.0786	0.0641	0.0311	0.0831
*t*	− 1.93	− 2.45	− 2.45	− 2.22	− 2.17	− 1.64	− 2.49
*p*	0.029	0.009	0.009	0.015	0.017	0.053	0.008

*T* test comparing the average 1951–1984 cold season temperatures with the average 1985–2016 cold season temperatures. The null hypothesis (Ho) is $\bar{\varvec{x}}$ (1951–1984) > (1985–2016). For all but one season, the Jan–Feb means, we must reject Ho (*p* < 0.05)

A similar pattern is obtained using minimum and maximum temperatures (Fig. S1a). Exceptionally cold central European winters coincide with insect population peaks in 1963 and 1981, whereas much warmer winters describe the period of ‘missing’ Alpine-wide epidemics since the mid-1980s. Particularly warm November–February conditions were recorded during the onsets of the three LBM outbreaks in 1989/1990, 1998 and 2007. All four outbreaks between 1953 and 1982 occurred during strong negative phases of the NAO (Fig. 3a), whereas positive NAO indices dominate the period of their disappearance (Table 2). Both the lowest winter temperature anomalies and the most negative NAO indices between 1950 and 2018 were recorded simultaneously in 1963, followed by 1953.

**Table 2:** LBM cycles and winter climates

	1953–1955	1962–1964	1971–1973	1980–1982	1989–1991	1998–2000	2007–2009	2017–2018
Min Temp	− 0.39	− 0.54	0.23	− 0.49	0.27	− 0.03	0.01	− 0.38
Mean Temp	− 0.31	− 0.59	0.16	− 0.51	0.37	− 0.02	0.16	− 0.15
Max Temp	− 0.28	− 0.51	0.09	− 0.49	0.45	− 0.07	0.42	0.04
NAO Index	− 0.47	− 1.26	− 0.01	− 1.01	0.36	0.13	0.01	− 0.48

Minimum, mean and maximum winter (Nov–Feb) temperature anomalies (°C with respect to 1981–2010), as well as winter NAO indices (Nov–Feb) averaged over each of the three-year periods of the past eight LBM cycles. Temperature data were extracted from E-OBS and averaged over 6–12° E and 46–47° N, and NAO indices have been obtained from NOAA’s Climate Prediction Center (CPC)

In addition to distinct year-to-year winter temperature variability since 1950, the most obvious feature is the regime shift from generally cooler winters before 1987 to mostly warmer conditions afterwards (Fig. 3b). With a temperature range from − 0.46 to 0.21 °C (Fig. S1b), all monthly winter combinations reveal substantial temperature differences between the cooler high-peak and warmer low-peak outbreak period. The recent Alpine winter cooling originates from a large-scale mode change over the North Atlantic/European sector. Known as the ‘Beast from the East’, exceptionally cold Arctic airmasses with an anticyclonic structure were stretching from Siberia to the British Isles, covering large parts of Asia and almost all of Europe from 24-Feb-2018 to the second half of March 2018. Apart from the remarkable agreement between cold winters and the occurrence of LBM outbreaks, no other season (e.g. summer), or meteorological parameter (e.g. precipitation), was found to be statistically related. Differences between cold November–February temperatures and relatively warm April conditions, however, reveal specifically large variances before and during the four LBM outbreaks that were recorded between 1953 and 1982, and again in 2018 (Fig. S1a). The three LBM cycles that were not associated with extensive population outbreaks between 1998 and 2009 were characterised by a preponderance of mild winters and overall small seasonal temperature differences.

## Discussion

Our study describes a remarkable, recent, eco-climatic phenomenon: nearly four decades ago, population and forest ecologists were aghast when the regular, clockwork-like cycles of landscape-scale defoliation caused by the LBM in the subalpine larch belt of the European Alps suddenly stopped. Based on the varying widths of precisely dated tree rings and their mean site chronologies, it was concluded that LBM outbreaks had persisted for more than 1200 years (Esper et al. 2007), so it was rather unexpected that intensive population peaks, and their subsequent forest defoliation and discoloration impact, failed to materialize after 1981. Despite much speculation about the apparent ‘collapse’ of LBM outbreaks (Büntgen et al. 2009; Esper et al. 2007; Hartl-Meier et al. 2017; Ims et al. 2008; Johnson et al. 2010; Kress et al. 2009; Nola et al. 2006; Rolland et al. 2001; Saulnier et al. 2017), the underlying ecological processes causing this change remained unresolved.

**Fig. 4:**
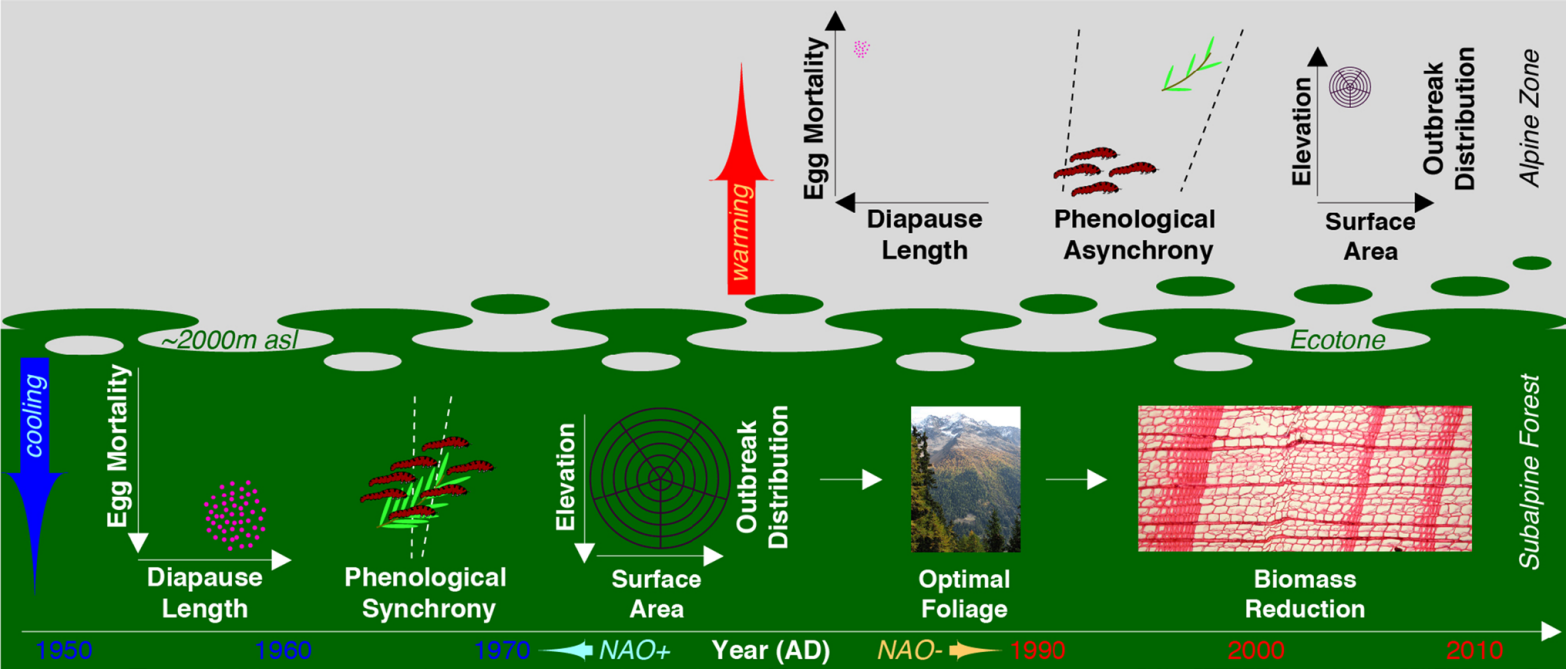
Effects of rising winter temperatures on LBM outbreaks. Associated with predominantly negative phases of the NAO, central Europe experienced relatively cold winters from the mid-twentieth century until around 1988. During this period three intertwined, temperature-induced mechanisms are proposed to explain the cyclic recurrence of four, high-peak, Alpine-wide LBM outbreaks in 1953–55, 1962–64, 1971–73, and 1980–82. (i) Reduced egg mortality due to lower energy consumption throughout extended diapause, (ii) phenological synchrony between egg hatching and larch needle growth, and (iii) location of extensive outbreak epicentre within dense subalpine larch forests. Optimal host needle foliage is expressed by a picture of the subalpine larch forest in the Lötschental, and LBM-induced biomass reduction is shown by the 1963 LBM outbreak that reduced both, tree-ring width and latewood density (example from the Lötschental; photos UB)

Another surprising chapter in this story, however, occurs now: In 2017 and 2018, LBM outbreaks returned across many areas after nearly four decades of disappearance. This unexpected return of outbreaks suggests that phase shifts in the NAO may also have shifted outbreak epicentres back into lower elevations where subalpine larch forests exist (Fig. 4). Furthermore, warm winters may have caused elevated respiration of LBM eggs during diapause leading to a decoupling of LBM with foliar development (Baltensweiler 1993), but recent cold winters may have reversed this trend. Nevertheless, it remains debatable if higher energy consumption during dormancy accelerates egg hatching towards earlier, non-optimal late winter conditions, for which intra- and interannual variation in snow fall and snow coverage add another level of complexity (Williams et al. 2015). Moreover, warmer winter and spring temperatures (together with increased energy stress) can trigger asynchrony between advanced egg hatch and the expansion of larch foliage (Benz 1974; Baltensweiler 1993; van Asch et al. 2012). Presently, the onset of larch needle growth tends to be earlier in the growing season than it was during most of the years when it was monitored before the mid-1980s (Fig. S2b). An early budburst in 1971, 1981 and 2018 synchronised hatching and initiation of needle growth. Substantial differences in the growth response to thermal thresholds, between insects and their host vegetation, however, lead to trophic asynchrony (Renner and Zohner 2018), so-called phenological mismatch between life cycle phases which, in the case of the LBM, ultimately reduce larval performance. The resulting disruption of antagonistic trophic interactions will only have negative impacts on LBM population growth. The ecological consequences of warming-induced diverging phenological phases may even increase with elevation (i.e. faster insect responses versus slower host responses) (Moser et al. 2010), a phenomenon well described in cold environments at high-northern latitudes (Ims et al. 2008; Post et al. 2009). Finally, warmer temperatures may possibly shift the location of optimal LBM growth conditions to higher elevations (Johnson et al. 2010). The theory of resource limitation, due to upward elevational shifts in LBM outbreak epicentres, into areas with limited larch foliar biomass, is corroborated by findings from the Bavarian pre-Alps (Hartl-Meier et al. 2017) and the Tatra Mountains in northern Slovakia (Büntgen et al. 2009; Konter et al. 2015), where widespread epidemics have neither been observed nor reconstructed, likely due to the lack of extensive larch forests. In addition to these proposed mechanisms, changes in land-use/land-cover and tree species composition might play an additional role in dampening LBM outbreak intensity via diminished foliage resources (Battipaglia et al. 2014).

Based on yearly larval counts and visual observation of defoliation in the eastern Swiss Alps (Grisons) (Wermelinger et al. 2018), as well as yearly counts of larval density in a permanent plot at 1800 m asl in the southern French Alps (Les Combes, Briançon) (Roques and Goussard 1982), our study—though limited in space and time—reveals that LBM population oscillations most likely did not stop during the last decades. This detracts from the postulated ‘collapse’ of cyclic population dynamics in response to climate change (Ims et al. 2008). Since some of the contemporary defoliation reports are from relatively high-altitudes between 1600 and 2000 m asl, they are indicative of outbreaks near the upper distributional limit of extensive larch stands. This finding corroborates predictions of temperature-mediated elevational shifts in LBM outbreak epicentres (Johnson et al. 2010). From the Little Ice Age until the mid-1980s, the optimal elevational range for LBM outbreaks was likely below the upper limit of extensive subalpine larch forests. Since the 1990s, the temperature sensitive LBM range though possibly exceeded the host resource distribution by moving upwards into the predominantly treeless Alpine zone. The recent return of LBM outbreaks does not exclude a downward shift in epicentres from above the ecotone back into the subalpine forest zone. Dendrochronological fingerprints of the four LBM outbreak peaks between 1953 and 1982 (Fig. 2j–k) suggest the current population boost will also cause abrupt reductions in the ring width and/or latewood density of its subalpine larch hosts. In line with dendrochronological findings from the French Alps (Saulnier et al. 2017), our tree-ring evidence from the Swiss Lötschental confirms the absence of strong outbreaks after 1982 (Fig. 2l), the period for which systematically collected, quantitative LBM data are limited for most of the Alpine arc, including Switzerland.

Our study argues for the often-neglected relevance of cold season climate for organismal responses (Williams et al. 2015; Büntgen and Krusic 2018), most pronounced at higher latitudes and elevations, where the impacts of cold season temperature and precipitation persist through most of the year. Trends and extremes in winter climate, which usually exceed those during other months (Renner and Zohner 2018), impact species-specific chilling requirements, the risk of frost injury, demands on energy and water balance, as well as phenological synchrony and the composition and interactions within communities (Williams et al. 2015; Renner and Zohner 2018). In addition to describing uneven rates of change in the phenology of interacting species, our results provide evidence of the persistence of LBM cycles during recent decades. While this finding contradicts arguments for a strong vulnerability of population cycles to climate (Ims and Fuglei 2005; Ims et al. 2008), it does not imply that transient population dynamics remain unaffected (i.e. although the system’s cyclic behaviour continued during the last decades, the amplitude of oscillations remained low; Fig. 2l). Instead, we argue for the importance of shifts in the NOA that can, at least sporadically, dampen or even reverse the ecological effects of increasing temperatures. Since internal climate oscillations play a critical role for cold season ecosystem functioning in central Europe (Stenseth et al. 2002), consideration of its teleconnection patterns is important for disentangling the effects of naturally and anthropogenically forced climate change (Rosenzweig and Neofotis 2013). In terms of ‘detection and attribution’—a modern principle in climate change research—improved understanding of the sensitivity of trophic interaction in biological and ecological systems to abiotic forcing factors will better inform and guide local to regional management strategies, national and international conservation programmes, and more generally, global environmental policy. Nevertheless, our study suggests the complexity of scale-dependent interactions between different, overlapping climate forcing agents. While a pause in recurring, geographically extensive LBM outbreaks after 1981 describes a response to anthropogenic global warming (Esper et al. 2007; Johnson et al. 2010; Saulnier et al. 2017), the recent return of a high LBM populations points towards the importance of synoptic-scale, internal climate variability versus externally forced, large-scale climate change (Stenseth et al. 2002). Unfortunately, our limited understanding of the spatiotemporal characteristics of LBM outbreaks prior to the mid-twentieth century hinders similar analyses of LBM dynamics further back in time.

In conclusion, tree-ring evidence from the Swiss Alps suggests that larch budmoth outbreaks have occurred every 8–9 years over the past 1200 years. The occurrence of Alpine-wide LBM outbreaks, however, ceased after 1982 but the underlying causes of this change have been uncertain. The sudden return of LBM outbreaks in 2017 and 2018 might be related to anomalous cold European winters due to a persistent negative phase of the NAO, which almost resembles conditions of the four consecutive insect outbreaks between 1953 and 1982. In contrast, three warming-induced mechanisms (1–3 or a combination thereof) help explain the disruption of LBM outbreaks: (1) high egg mortality, (2) asynchrony between egg hatch and foliage growth, and (3) upward shifts of outbreak epicentres. Although we demonstrate that the LBM cycle did not stop during the past decades, our findings stress the climate sensitivity of transient population dynamics. By providing a strong negative relationship between winter temperatures and LBM outbreaks, this study not only overcomes previous speculations, but also underlines the ecophysiological importance of separating natural from anthropogenic climate forcing of trophic interactions.

## Electronic supplementary material

Below is the link to the electronic supplementary material.

Supplementary material 1 (DOCX 2680 kb)
