# Supplementary material for: Return of the moth: rethinking the effect of climate on insect outbreaks
Source: Oecologia. 2020 Jan 9;192(2):543–52. doi: 10.1007/s00442-019-04585-9 (PMC7002459; doi:10.1007/s00442-019-04585-9)
Supplement: Supplementary file 1 — Supplementary material 1 (DOCX 2680 kb) [file 442_2019_4585_MOESM1_ESM.docx]

**Electronic Supplementary Material (ESM)**


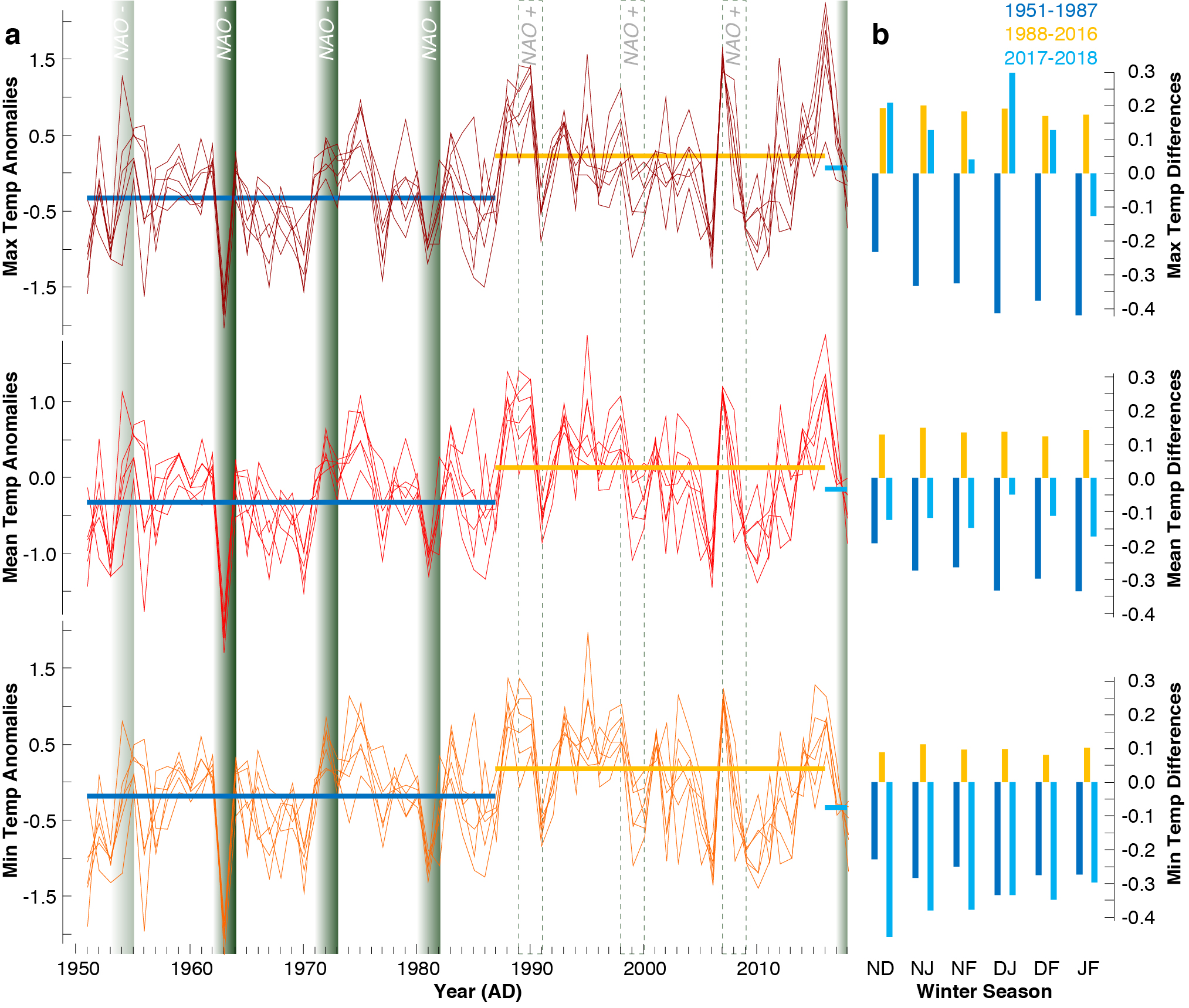


**Fig. S1** Temperature variability and LBM activity. a, Comparison between six different combinations of monthly minimum (orange), mean (red) and maximum (dark red) winter temperatures between November and February (similar to Fig. 2a). b, Temporal differences in Alpine winter temperatures between periods of intense and absent LBM outbreaks (similar to Fig. 2b).


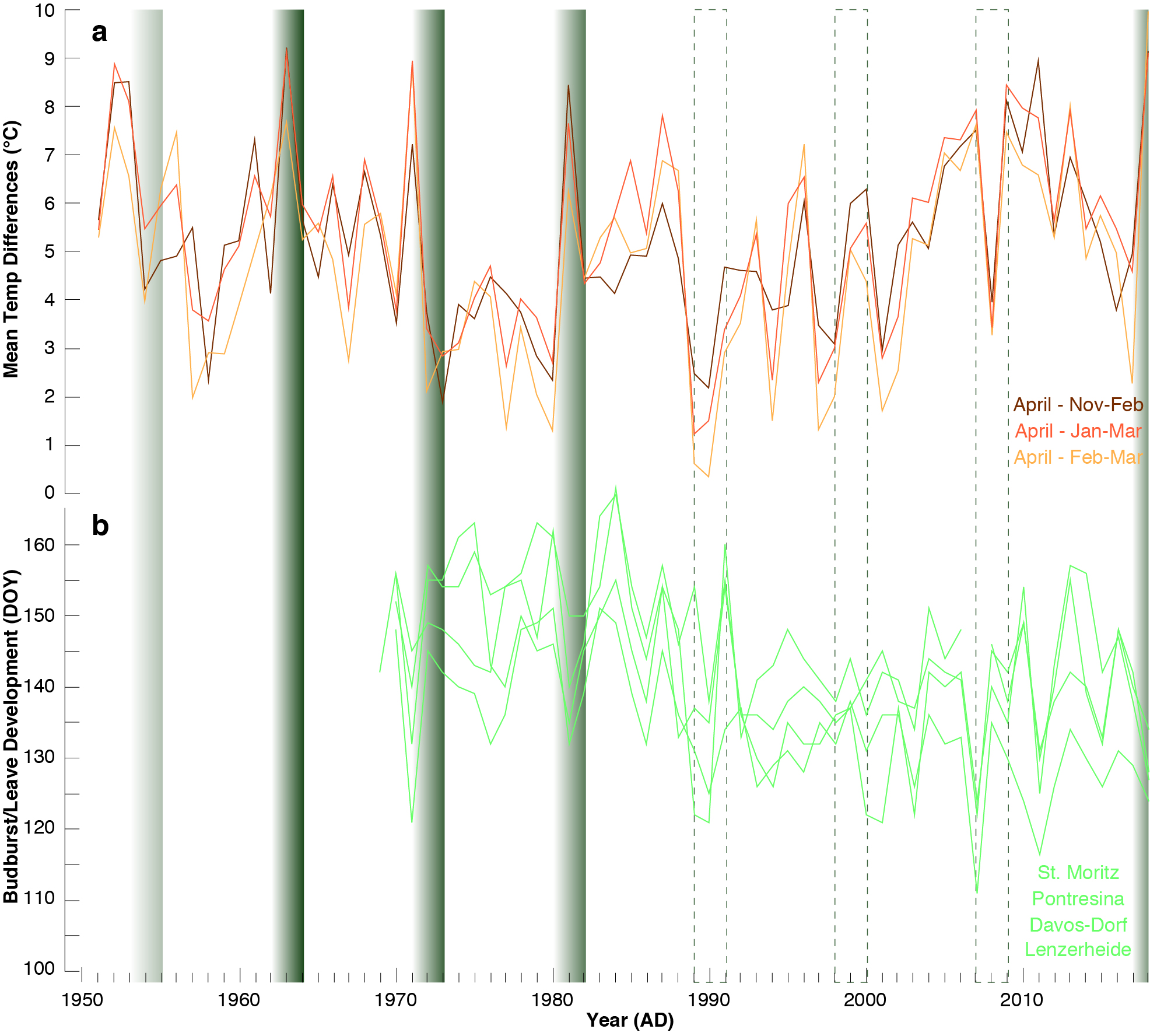


**Fig. S2** Seasonal temperature difference and host phenology. a, Differences between April and winter/spring (Nov–Dec, Jan–Mar and Feb–Mar) mean temperatures (extracted from E-OBS and averaged over 6–12° E and 46–47° N). b, Continuous phenological observations of the onset of needle growth at four subalpine larch sites in the eastern Swiss Alps between 1500 and 1800 m asl, expressed as day of the year (DOY).
